# Mitochondria-targeting multifunctional nanoplatform for cascade phototherapy and hypoxia-activated chemotherapy

**DOI:** 10.1186/s12951-022-01244-9

**Published:** 2022-01-21

**Authors:** Jie Lv, Shuangling Wang, Duo Qiao, Yulong Lin, Shuyang Hu, Meng Li

**Affiliations:** College of Pharmacy, Key Laboratory of Innovative Drug Development and Evaluation, Hebei Medical University, Shijiazhuang, 050017 China

**Keywords:** Mitochondria-targeting, Hypoxia-activated prodrug, Phototherapy, Combination therapy, Drug delivery

## Abstract

Despite considerable progress has been achieved in hypoxia-associated anti-tumor therapy, the efficacy of utilizing hypoxia-activated prodrugs alone is not satisfied owing to the inadequate hypoxia within the tumor regions. In this work, a mitochondrial targeted nanoplatform integrating photodynamic therapy, photothermal therapy and hypoxia-activated chemotherapy has been developed to synergistically treat cancer and maximize the therapeutic window. Polydopamine coated hollow copper sulfide nanoparticles were used as the photothermal nanoagents and thermosensitive drug carriers for loading the hypoxia-activated prodrug, TH302, in our study. Chlorin e6 (Ce6) and triphenyl phosphonium (TPP) were conjugated onto the surface of the nanoplatform. Under the action of TPP, the obtained nanoplatform preferentially accumulated in mitochondria to restore the drug activity and avoid drug resistance. Using 660 nm laser to excite Ce6 can generate ROS and simultaneously exacerbate the cellular hypoxia. While under the irradiation of 808 nm laser, the nanoplatform produced local heat which can increase the release of TH302 in tumor cells, ablate cancer cells as well as intensify the tumor hypoxia levels. The aggravated tumor hypoxia then significantly boosted the anti-tumor efficiency of TH302. Both in vitro and in vivo studies demonstrated the greatly improved anti-cancer activity compared to conventional hypoxia-associated chemotherapy. This work highlights the potential of using a combination of hypoxia-activated prodrugs plus phototherapy for synergistic cancer treatment.

**Graphical Abstract:**

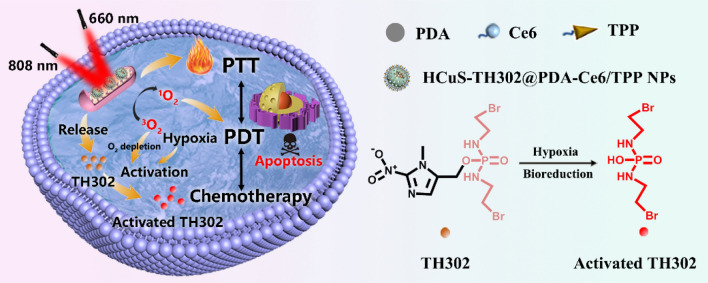

**Supplementary Information:**

The online version contains supplementary material available at 10.1186/s12951-022-01244-9.

## Introduction

Despite considerable progress has been made in cancer chemotherapy in the past few decades, the serious side effects that arise from the off-target actions and low pharmaceutical selectivity of the therapeutic agents still limit the clinical outcomes [1, 2]. Developing prodrugs sensitive to the tumor microenvironment (TME) would be a crucial subject for tumor-targeted chemotherapy. Tumor hypoxia which caused by the overwhelming oxygen consumption of tumor cells and the inadequate oxygen supply has been recognized as a typical feature of most solid tumors [3–5]. To this aim, hypoxia-activated prodrugs (HAPs) that only exert their diagnostic and therapeutic effect in tumor-specific hypoxic regions while keep in stealth at normal tissues have been intensively explored to against cancer [6, 7]. Especially, evofosfamide (TH302) as a clinical-stage HAP has received tremendous attention [8, 9]. Although promising, no positive phase III clinical results have been achieved, since the tumor regions nearby tumor vasculatures are still normoxia [10–12]. Thereby, inhibiting the oxygen supply or enhancing oxygen consumption in the tumor sites would be a potential strategy to improve the therapeutic efficiency of the TH302.

Photodynamic therapy (PDT) has aroused great interests recently due to its unique characteristics including high therapeutic efficacy, spatiotemporal controllability, and minimal invasiveness [13, 14]. PDT relies on a photosensitizer (PS) that generates reactive oxygen species (ROS) under photo irradiation to induce tumor cell apoptosis [15]. This process consumes oxygen and exaggerates tumor hypoxia [16, 17]. The exacerbated hypoxia in tumor cells can facilitate the activation of HAPs to improve their therapeutic effects. Therefore, combination of HAPs with PDT can be a promising approach to potentiate the anticancer efficiency [18–20]. However, the therapeutic efficiency of PDT is restricted by the narrow diffusion area and short lifespan of the cytotoxic ROS [21, 22]. Mitochondrion is the most primary organelle to generate ROS in living cells which plays a central role in oxidative metabolism and cell apoptosis, making it an ideal target for PDT [23, 24]. Moreover, TH302 exerts its action by releasing the DNA crosslinking agent bromo-isophosphoramide mustard (Br-IPM) in hypoxic tissue, which causes DNA damage and then cytotoxicity [12, 25]. However, this kind of drugs can easily develop drug resistance due to efficient DNA repair mechanisms [26]. Rerouting these drugs to the mitochondrion has been found to restore drug activity and bypass several drug resistance mechanisms [27]. Hence, designing mitochondrial targeted PDT/hypoxia-activated therapy would be an efficient strategy to enhance the anticancer activity. However, to the best of our knowledge, hypoxia activated mitochondria targeted nanoplatform has been rarely reported.

In addition to PDT, photothermal therapy (PTT) is another type of phototherapy used for cancer treatment that can destroy tumor cells via local hyperthermia generated by the photothermal agent upon photo irradiation [28]. Recently, it has been reported that PTT can also amplify the hypoxia of TME [7, 29]. The aggravated hypoxia would further trigger the activation of TH302. Moreover, by combination of PTT and PDT in one system, the ROS produced in the photodynamic process can suppress the expression of genes involved in heat resistance, thereby enhancing the sensitivity of the cancer cells to PTT [30, 31]. Compared with the conventional monomodal phototherapy, the synergistic PDT and PTT can significantly enhance the therapeutic efficiency for malignant carcinomas [32].

**Fig. 1:**
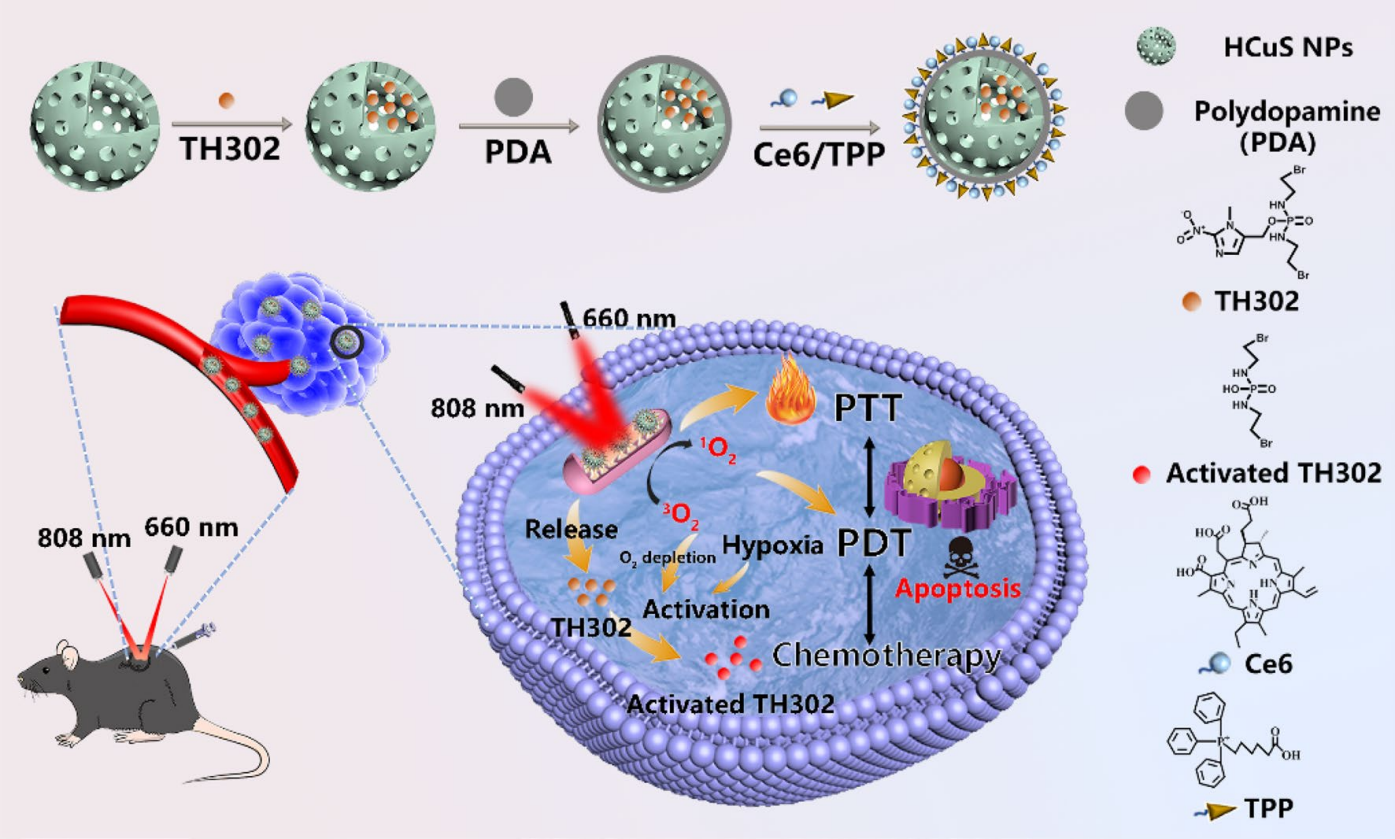
Schematic illustration of the preparation and therapeutic functions of HCuS-TH302@PDA-Ce6/TPP NPs

In the present study, we report the rational design of a mitochondrial targeted nanoplatform, denoted as HCuS-TH302@PDA-Ce6/TPP NP, which combines PDT, PTT and hypoxia-activated chemotherapy to synergistically treat cancer and maximize the therapeutic window. As demonstrated in Fig. 1, hollow copper sulfide nanoparticles (HCuS NPs) with high near-infrared (NIR) photothermal conversion efficiency were used as the photothermal nanoagents in our study. Importantly, the hollow and mesoporous structure endows HCuS NPs with high drug loading capacity [33, 34], making them as NIR responsive TH302 carriers for chemotherapy/photothermal synergistic therapy. Subsequently, polydopamine (PDA) was employed to encapsulate the HCuS NPs core. The PDA coating can not only act as a smart photothermal sensitive gatekeeper, but also serve as a substrate for the conjugation with a NIR excitable PDT agent and a mitochondria-targeting ligand. Chlorin e6 (Ce6) was chosen as the photosensitizer due to its activation by NIR light, relatively high quantum yield of ROS and good biocompatibility [35, 36]. While, triphenyl phosphonium (TPP) with the strong lipophilic and delocalized cationic nature was ulitized as the mitochondrial targeting moiety to target the inner mitochondrial membrane especially in tumor cells [37, 38]. On the basis of this, the obtained nanoplatform preferentially accumulated in mitochondria under the action of TPP. Using 660 nm laser to excite Ce6 can generate ROS and simultaneously exacerbate the hypoxia in the tumor cells. While under the irradiation of 808 nm laser, the nanoplatform produced local heat which can increase the release of TH302 in tumor cells, ablate cancer cells as well as aggravate the hypoxia of TME. Sequential irradiation with two different wavelengths also enabled the stepwise activation of PDT and PTT, maximizing therapeutic efficacy through a synergistic manner [39]. As expected, benefiting from the localized therapeutic effect of PDT/PTT and hypoxia-activated cytotoxicity of TH302, this nanoplatform exhibited enhanced therapeutic efficacy with negligible systemic toxicity both in vitro and in vivo.

## Materials and methods

### Chemicals and materials

Copper chloride (CuCl_2_·2H_2_O), polyvinylpyrrolidone (PVP-K30), hydrazine anhydrous solution (N_2_H_4_·H_2_O), dopamine hydrochloride, (5-carboxypentyl) triphenylphosphonium bromide (TPP), tris(hydroxymethyl)aminomethane (Tris), *N*-hydroxysuccinimide (NHS) were purchased from Aladdin. Sodium sulphide (Na_2_S·9H_2_O) was obtained from Tianjin Yongda Chemical Reagent Co., Ltd. Sodium hydroxide (NaOH) was purchased from Tianjin Fengchuan Chemical Reagent Technology Co., Ltd. Chlorin e6 (Ce6) was purchased from Frontier Scientific. 1-(3-dimethylaminopropyl)-3-ethylcarbodiimide hydrochloride (EDC) was purchased from J&K Scientific. 2′,7′-dichlorofluorescein diacetate (DCFH-DA) and Mito-Tracker Green were purchased from Beyotime Biotechnology. 3-(4,5-dimethylthiazol-2-yl)-2,5-diphenyltetrazolium bromide (MTT) was obtained from Beijing Innochem Co. Ltd. DAPI (4′,6-diamidino-2-phenylindole) was bought from Sigma-Aldrich. All the reagents were of analytical grade and directly used without additional purification. Deionized water was obtained from a Millipore water purification system.

### Instruments

High resolution transmission electron microscopy (HRTEM) images were obtained using a JEM-2100 transmission electron microscope (JEOL Ltd., Japan) operated at 120 kV. Powder X-ray diffraction (XRD) patterns were performed on a Rigaku D/max-2500 X-ray diffractometer with CuKα radiation (Japan). The Brunauer–Emmett–Teller (BET) surface area and total pore volume were calculated from N_2_ adsorption–desorption isotherms that were recorded on a Micromeritics ASAP 2020 M automated sorption analyzer. The UV-vis absorption spectra were recorded on a PERSEE T-9 (Beijing Persee General Instrument Co. Ltd., China). The fluorescence spectra were acquired on a HITACHI F-7100 fluorescent spectrophotometer. The sizes and zeta potentials of nanomaterials were measured by a 90 plus PALS particle size analyzer (Brookhaven, America). Fourier translation infrared (FTIR) spectra were obtained on a SHIMADZU FTIR-8400S spectrophotometer (Shimadzu, Japan) using KBr pellet method. The drug concentration was determined by high performance liquid chromatography (HPLC) on an Agilent. Fluorescence images were conducted using a confocal laser scanning microscope (CLSM, Leica TCS SP5). Elemental analysis was performed on an inductively coupled plasma-atomic emission spectrometer (ICP-AES, Shimadzu ICPE-9000).

### Preparation of HCuS NPs

HCuS NPs were synthesized according to previously reported method with minor modification [40]. Briefly, 100 μL CuCl_2_ solution (0.5 M) was added to 25 mL deionized water containing 0.24 g PVP-K30 under magnetic stirring at room temperature. Then, 25 mL NaOH solution (pH = 9.0) was added, followed by addition of 6.4 μL N_2_H_4_·H_2_O (50%) to form a bright-yellow suspension of Cu_2_O spheres. After 5 min, 200 μL Na_2_S aqueous solution (320 mg mL^−1^) was added to the suspension. The solution was heated for 2 h at 60 °C. Finally, HCuS NPs were centrifuged at 12,000 rpm for 8 min, and washed three times with deionized water and ethanol respectively. Then, the product was dried under vacuum at 60 °C overnight.

### Synthesis of PDA-modified HCuS (HCuS@PDA NPs)

Coating PDA onto the surface of HCuS NPs was realized via the oxidative self-polymerization of dopamine under alkaline conditions [41]. Briefly, HCuS NPs (1 mg) and dopamine hydrochloride (1.2 mg) were added in turn into 10 mL Tris solution (10 mM, pH = 8.5), followed by intensive stirring for 12 h at room temperature. Then the resulting HCuS@PDA NPs were collected by centrifugation (12,000 rpm, 8 min), and washed with deionized water thrice. Finally, the product was dried under vacuum at 60 °C overnight.

### Preparation of HCuS@PDA-Ce6/TPP NPs

1 mg of Ce6 was dissolved in DMSO (1 mL). Then, EDC and NHS were added to activate the carboxyl groups of Ce6 for 3 h [42]. On the other hand, TPP (3 mg) was also dissolved in DMSO (1 mL) and activated by EDC/NHS for 3 h [43]. After that, the above solutions were mixed and added to the HCuS@PDA NPs suspension (1 mL, 1 mg mL^−1^) under stirring for 24 h. The crude products were collected by centrifugation (12,000 rpm, 8 min), and washed thrice with deionized water to obtain HCuS@PDA-Ce6/TPP NPs. Then, the product was dried under vacuum at 60 °C overnight.

### Photothermal properties of HCuS based nanomaterials

Aqueous dispersion (1 mL) containing HCuS or HCuS@PDA NPs nanoparticles was exposed to NIR laser irradiation (808 nm) for 8 min. Real-time temperature of different samples was measured every 30 s using a FLIR infrared camera during the course. NIR laser power dependent temperature elevation was also measured in accordance with the same procedure.

### Photodynamic properties of HCuS@PDA-Ce6/TPP NPs

Briefly, 30 μL DPBF solution (0.75 mg mL^−1^) in ethanol was added into aqueous dispersion of Ce6 or HCuS@PDA-Ce6/TPP NPs (3 mL, equivalent Ce6 dosage: 4 μg mL^−1^) under gently mixing. Afterwards, the mixture was irradiated by the 660 nm laser (0.3 W cm^−2^, 30 min). UV–vis absorption spectra of the samples were monitored at various time points during laser irradiation, and the absorbance decay at 410 nm was calculated.

### Drug loading and release profile measurement

The typical drug loading process was performed as follows: HCuS NPs (10 mg) was dispersed in TH302 aqueous solution (300 μM, 10 mL). Then the mixture was stirred for 12 h, which was followed by centrifugal operation at 12,000 rpm for 8 min. The supernatant was extracted and the concentration of TH302 in the supernatant was determined by high performance liquid chromatography (HPLC). The Venusil ASB C18 column (4.6 mm × 150 mm, 5 μm) was used. The mobile phase was consisted of acetonitrile–water (70:30, V/V) with a flow rate of 1 mL min^−1^, and the detection wavelength was 316 nm. The column temperature was 30 °C and the injection volume was 10 μL.

The real-time drug release profile of TH302 from HCuS-TH302@PDA NPs was determined at pH 5.0 and pH 7.4 in PBS, respectively. The HCuS-TH302@PDA NPs was dispersed in PBS solution (pH 7.4 or 5.0) under magnetic stirring at 37 °C. And at 2 h, 4 h time points, the suspension at pH 5.0 or 7.4 was irradiated with 808 nm laser (0.5 W cm^−2^) for 5 min to evaluate the photothermal effect on TH302 release. 200 μL of suspension was taken at time intervals of 0 h, 0.5 h, 1 h, 2 h, 2.083 h, 3 h, 4 h, 4.083 h, 6 h, 12 h and 24 h, respectively, and then centrifuged to get the supernatant. After that, the supernatant was detected by HPLC and the amount of the released TH302 was calculated via the peak area.

### Fluorescence imaging

B16F10 cells were cultured in RPMI 1640 medium with 1% penicillin–streptomycin solution and 10% fetal bovine serum (FBS, BI) at 37 °C in 5% CO_2_. B16F10 cells were cultured in 24-well plates (2 × 10^4^ cells/well) and incubated at the normoxic environment. After 24 h, the culture medium was replaced with fresh medium containing HCuS@PDA-Ce6/TPP NPs (50 μg mL^−1^) and the cell was incubated for 4 h. Then, the cells were rinsed with PBS, and incubated with Mito-Tracker Green (5 nM) for 30 min, fixed by 4% paraformaldehyde for 30 min and stained with DAPI (1 μg mL^−1^) for 30 min. The cellular uptake and co-localization experiments were examined by CLSM.

### In vitro ROS/hypoxia assay

The B16F10 cells (3 × 10^4^ cells/well) seeded on 24-well plates were cultured at 37 °C with 5% CO_2_ for 24 h. Then, the cells were co-incubated with HCuS@PDA-Ce6/TPP NPs (0, 10, 25 and 50 μg mL^−1^) for 4 h. Then the cells were washed with PBS three times and further incubated with ROS fluorescent probe (DCFH-DA, final concentration of 10 μM) in RPMI 1640 medium (free FBS) for 30 min. Afterward, the cells were washed three times and irradiated with 660 nm laser for 5 min (0.3 W cm^−2^). The fluorescence of DCF was measured by fluorescence spectrophotometer with the same live cell numbers for each sample.

For cellular hypoxia analysis, the B16F10 cells (3 × 10^4^ cells/well) seeded on 24-well plates were cultured at the normoxic condition for 24 h. After that, the cells were treated with HCuS@PDA-Ce6/TPP NPs (50 μg mL^−1^) for 4 h. Then the cells were washed with PBS three times and then irradiation for 5 min with 660 nm laser irradiation (0.3 W cm^−2^) in fresh culture medium. Afterward, the cells were incubated with pimonidazole hydrochloride (Hypoxyprobe-1™ plus kit, Hypoxyprobe, Burlington, MA) for 60 min. The cells were washed with PBS and stained by FITC-Mab1 and DAPI for fluorescence microscope observation.

### Cytotoxicity assay

To evaluate the cell cytotoxicity of HCuS@PDA-Ce6/TPP NPs, B16F10 cells seeded on the 96-well plates were cultured at 37 °C with 5% CO_2_ for 24 h. Next, the cells were treated with HCuS@PDA-Ce6/TPP NPs (0, 1, 2.5, 5, 10, 25 and 50 μg mL^−1^). After incubation for 24 h, standard MTT assay was used to detect the cell viability. The absorbance values of formazan were determined at 570/630 nm with a microplate reader.

In vitro phototherapy experiments were performed using MTT assay to determine the optimum conditions of irradiation. For PDT, B16F10 cells pre-seeded in 96-well plates were treated with HCuS@PDA-Ce6/TPP NPs at different concentrations (0, 5, 10, 25 and 50 μg mL^−1^) for 4 h. And then the cells were exposed to 660 nm laser with a low laser power (0.3 W cm^−2^) for 5 min and cultured at 37 °C for another 20 h. The standard MTT assay was used to detect the cell viability. Using the same method, the PTT performance was tested with 808 nm laser at 0.5 W cm^−2^ and 1.0 W cm^−2^.

The synergistic therapeutic effects of HCuS@PDA-Ce6/TPP NPs were also assessed by MTT assay. B16F10 cells pre-seeded in 96-well plates were treated with TH302 (23.86 μM, the concentration was equivalent to that of TH302 loaded in HCuS-TH302@PDA-Ce6/TPP NPs), HCuS@PDA-Ce6/TPP NPs (25 μg mL^−1^) or HCuS-TH302@PDA-Ce6/TPP NPs (25 μg mL^−1^) under hypoxic (1% O_2_, 5% CO_2_) or normoxic (21% O_2_, 5% CO_2_) conditions for 4 h. And then the cells of phototherapy groups were irradiated with lasers (660 nm, 0.3 W cm^−2^, 5 min; 808 nm, 1.0 W cm^−2^, 5 min) and cultured at 37 °C for another 20 h. The cell viability was detected by MTT assay.

### Hemolysis assay

First, whole blood was taken from the orbital venous plexus of SD rats (purchased from Laboratory Animals Center of the Hebei Medical University) and red blood cells (RBCs) were centrifugally isolated at 4000 rpm for 10 min. After purification with PBS for five times, RBCs (0.8 mL, 5% v/v) were mixed with PBS containing HCuS NPs, HCuS@PDA NPs and HCuS@PDA-Ce6/TPP NPs at multiple concentrations (1, 5, 10, 25, 50 and 100 μg mL^−1^), and the mixture was incubated at 37 °C for 2 h. The PBS and deionized water were considered as negative (0% hemolysis) and positive controls (100% hemolysis), respectively. Then, the supernatant was obtained by centrifuging the mixtures at 4000 rpm for 10 min and the absorbance at 570 nm was determined by UV–vis absorption spectra. The percent of hemolysis was calculated using the follow equation

1 $${\text{Hemolysis}}\left( \% \right) = \left( {A_{sample} - A_{negative} } \right)/\left( {A_{positive} - A_{negative} } \right) \times {1}00$$

where *A*_*sample*_ stands for the absorbance of the samples, and *A*_*negative*_ and *A*_*positive*_ indicate the absorbance of the negative and the positive control samples, respectively.

### In vivo biocompatibility

Female C57BL/6 mice (6 weeks, 18–20 g) were purchased from Laboratory Animals Center of the Hebei Medical University. All the animal handing protocols and procedures were performed following the guidelines of the Hebei Committee for Care and Use of Laboratory Animals, and were approved by the Animal Experimentation Ethics Committee of the Hebei Medical University. Mice were kept in a sterile environment with free access to food and water. Tumor-free female C57BL/6 mice (20–25 g) were randomly divided into a control group and a HCuS@PDA-Ce6/TPP NPs group for in vivo biocompatibility experiment (6 mice per group). At day 0, mice treated with PBS (control group) and HCuS@PDA-Ce6/TPP NPs (2 mg mL^−1^) through intravenous injection, respectively. The body weight of each mouse was recorded every day. After 21 days, the mice were sacrificed, and the main organs (hearts, livers, spleens, lungs, and kidneys) were excised for tissue section and imaging.

### In vivo antitumor efficiency of HCuS@PDA-Ce6/TPP NPs

Female C57BL/6 mice (6 weeks, 18–20 g) were purchased from Laboratory Animals Center of the Hebei Medical University. To establish tumor models, 100 μL of B16F10 cells (1.0 × 10^7^ per mL) were subcutaneously inoculated in the right dorsal region. After that, all the mice were bred till the tumor volume of 80–100 mm^3^ was attained. The tumor volume was calculated according to the following formula: tumor volume (mm^3^) = 1/2 × length × width^2^. To investigate the anti-tumor effect in vivo, tumor bearing mice were randomly divided into seven groups (n = 7 each group), and treated as follows: (1) PBS, (2) PBS + 660 nm + 808 nm laser, (3) HCuS@PDA-Ce6/TPP NPs, (4) TH302, (5) HCuS-TH302@PDA-Ce6/TPP NPs, (6) HCuS@PDA-Ce6/TPP NPs + 660 nm + 808 nm laser, (7) HCuS-TH302@PDA-Ce6/TPP NPs + 660 nm + 808 nm laser. The mice were intratumorally injected with PBS (100 μL), HCuS@PDA-Ce6/TPP NPs (5 mg kg^−1^) and TH302 (0.2 mg kg^−1^) on days 0. After 4 h injection, the mice of groups (2), (6), and (7) were exposed to laser (660 nm, 0.3 W cm^−2^, 5 min; 808 nm, 1.0 W cm^−2^, 5 min). The tumor volumes and body weights of the mice were monitored every day. After 10 days, the mice were sacrificed, and the tumors were excised for tissue section and imaging. And the tumor growth inhibition rate (TGI %) was determined using the equation

2 $${\text{TGI}}\% = (V_{c} - V_{t} )/(V_{c} - V_{o} ) \times {1}00$$

where *V*_*c*_ and *V*_*t*_ are the mean tumor volumes of control and treated groups on day 10 and *V*_*o*_ is the mean tumor volume at the start of the study.

### In vivo biodistribution

The B16F10 tumor-bearing C57BL/6 mice were randomly divided into two groups (4 mice per group) and treated with PBS (control group) and HCuS@PDA-Ce6/TPP NPs (20 mg kg^−1^) through intravenous injection, respectively. After 4 h and 8 h, mice were sacrificed, and the organs (heart, liver, spleen, lung, kidney and tumor) were extracted. The Cu content of the samples was detected by ICP-AES.

### Statistical analysis

All data were presented as mean ± standard deviation. The statistical significance was determined using one-way analysis of variance (ANOVA). Significant differences between groups were indicated by *P < 0.05, **P < 0.01 and ***P < 0.001, respectively.

## Results and discussion

### Synthesis and characterization of HCuS@PDA-Ce6/TPP NPs

**Fig. 2:**
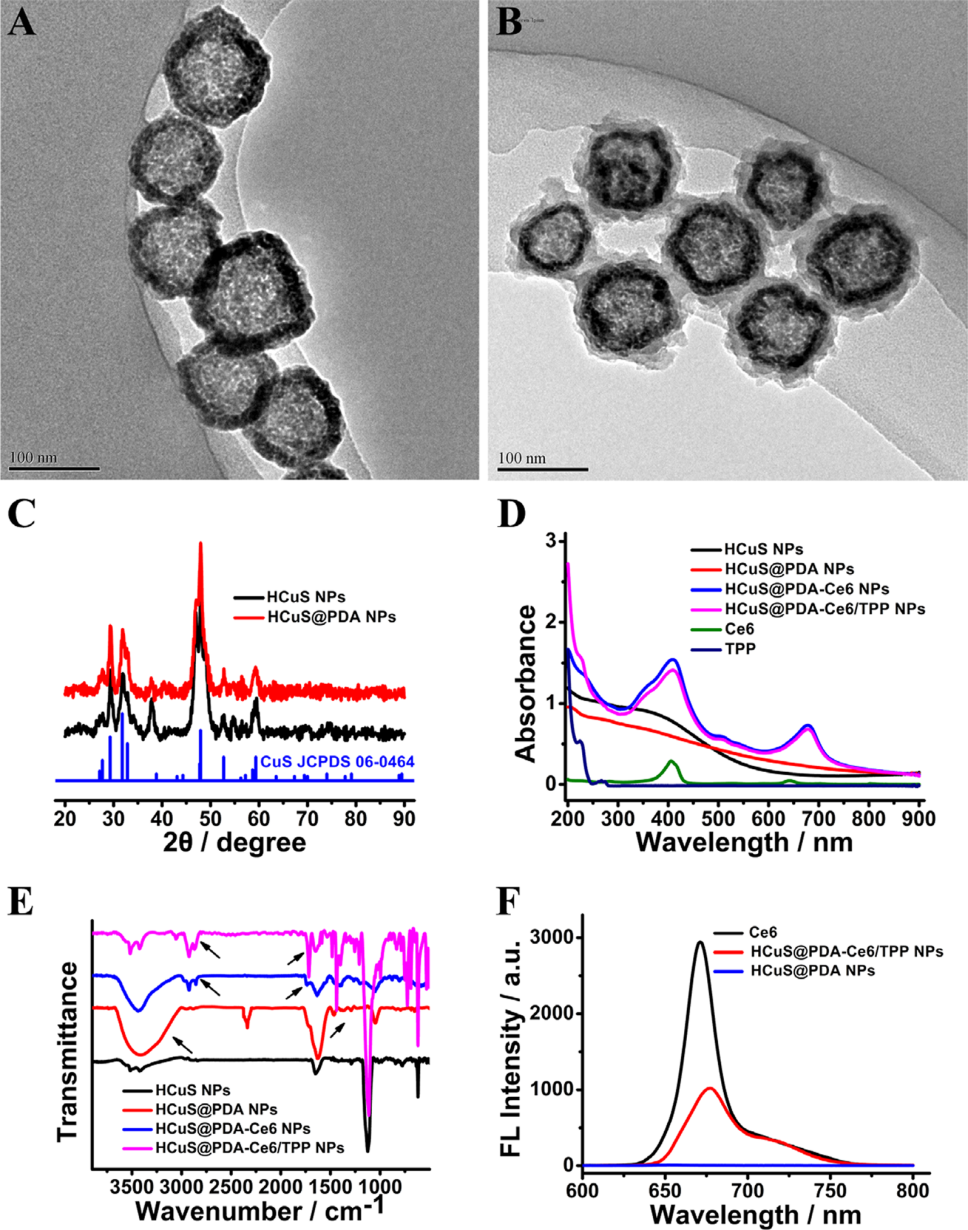
Characterization of HCuS-based nanomaterials. **A** TEM image of HCuS NPs. **B** TEM image of HCuS@PDA NPs. **C** XRD patterns of HCuS NPs and HCuS@PDA NPs. **D** UV–vis-NIR spectra of HCuS-based nanomaterials. **E** FTIR spectra of HCuS-based nanomaterials. **F** Fluorescence spectra of HCuS-based nanomaterials

HCuS NPs were synthesized via a one-pot sacrificial template method [40]. Transmission electron microscopy (TEM) image showed the synthesized HCuS NPs had an average diameter of around 100 nm with hollow interiors (Fig. 2A). As indicated in SEM image, the obtained nanoparticles were uniform with weak agglomeration (Additional file 1: Fig. S1A). The diffraction peaks in the XRD patterns of HCuS NPs (Fig. 2C) were indexed to the hexagonal phase of CuS (JCPDS No. 06-0464) [40]. The N_2_ adsorption–desorption measurements showed the surface area and pore volume were 14.37 m^2^ g^−1^ and 0.12 cm^3^ g^−1^, respectively (Additional file 1: Fig. S2), which validated the mesoporous features of HCuS NPs. The mesoporous nature endowed the HCuS NPs with the ability to load HAPs for cancer therapy [34, 40]. In addition, HCuS NPs possessed a broad NIR absorption capacity, suggesting their pronounced photothermal potential (Fig. 2D).

Coating PDA onto the surface of HCuS NPs was realized via the oxidative self-polymerization of dopamine under alkaline conditions [41]. The TEM image (Fig. 2B) and SEM image (Additional file 1: Fig. S1B) revealed that after coated with PDA, there was no clear difference in the shape of the HCuS NPs. But a smooth layer was formed on the surface of HCuS NPs and the mean thickness of the PDA coating shell was 15 nm (Fig. 2B). Furthermore, the surface charge of HCuS NPs was changed from − 11.70 ± 0.62 mV to − 28.55 ± 0.35 mV after coating with PDA (Additional file 1: Fig. S3). Besides, dynamic light scattering (DLS) analysis (Additional file 1: Fig. S4) proved that after PDA coating, the size of the nanoparticles gradually increased from 154.57 ± 2.96 nm (HCuS NPs) to 204.87 ± 2.57 nm (HCuS@PDA NPs). The XRD data indicated that the introduction of PDA could not disturb the structure of HCuS NPs (Fig. 2C). The successful modification was also validated by the appearance of the broad absorbance bands at 3500 cm^−1^ to 3000 cm^−1^ in FTIR spectra, which belonged to the O–H stretching and N–H stretching in the hydroxyl and amino groups of PDA (Fig. 2E). Meanwhile, the typical bands of benzene ring skeleton vibration bands at 1600 cm^−1^ to 1100 cm^−1^ were also observed (Fig. 2E). All above characteristics demonstrated that the PDA layer was successfully coated on the surface of HCuS NPs. Quantification of the density of PDA on the surface of HCuS NPs was accomplished by thermogravimetric analysis (TGA) (Additional file 1: Fig. S5), which corresponded to about 475 µg mg^−1^ HCuS@PDA NPs.

After that, Ce6 and TPP were covalently conjugated to the surface of HCuS@PDA NPs. The successful conjugation of Ce6/TPP was confirmed by UV–vis-NIR spectra, fluorescence spectra and FTIR spectra. Compared with HCuS@PDA NPs, HCuS@PDA-Ce6/TPP NPs exhibited two new absorption peaks in the UV–vis-NIR spectra at 413 and 676 nm, corresponding to the characteristic Soret and Q bands of Ce6 molecules (Fig. 2D) [44]. In addition, a shoulder peak at about 223 nm appeared in the spectrum of HCuS@PDA-Ce6/TPP NPs, which might be caused by the TPP moiety. As shown in the FTIR spectroscopy of HCuS@PDA-Ce6/TPP NPs (Fig. 2E), a peak at 2927 cm^−1^ occurred and the peak at 1730 cm^−1^ enhanced in intensity, which could be attributed to the stretching vibration of alkyl C–H and C=O from Ce6 or TPP, respectively. Conjugation onto the surface of HCuS@PDA NPs caused the emission peak of Ce6 to red-shift from 670 to 677 nm (Fig. 2F). In addition, the fluorescence intensity of HCuS@PDA-Ce6/TPP NPs was much weaker than that of free Ce6 at the same concentration, which should be attributed to the overlap between the absorption band of HCuS@PDA NPs and the emission band of Ce6 (Fig. 2F). Moreover, conjugating Ce6/TPP to the surface of nanoparticles could not change the morphology of the HCuS@PDA NPs, which was revealed by the TEM images and SEM images (Additional file 1: Fig. S1, S6). The DLS data showed that the average sizes of HCuS@PDA-Ce6 NPs and HCuS@PDA-Ce6/TPP NPs were 254.15 ± 2.89 nm and 257.62 ± 2.84 nm, respectively (Additional file 1: Fig. S4). All the results indicated Ce6 and TPP have been conjugated onto the surface of the nanoparticles.

**Fig. 3:**
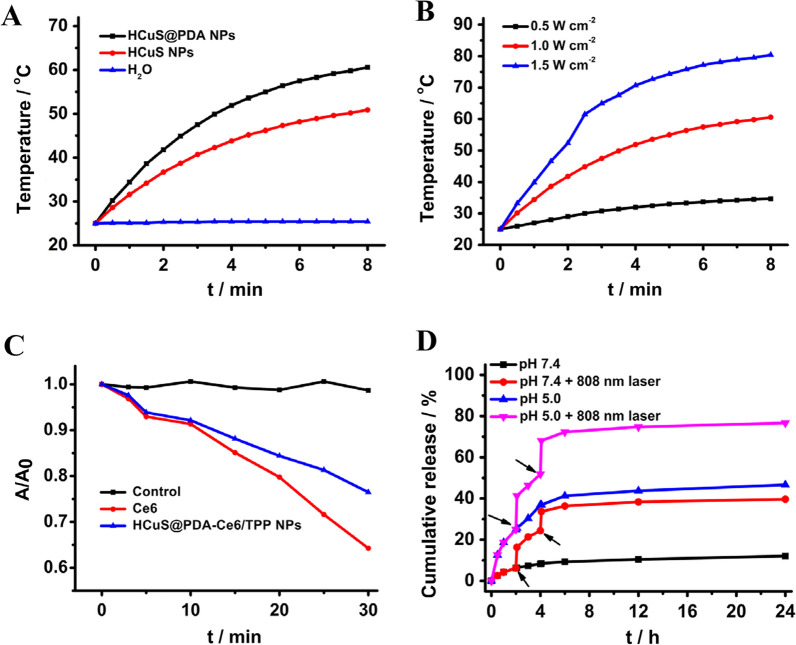
**A** Photothermal response of HCuS NPs and HCuS@PDA NPs upon laser irradiation (808 nm, 1.0 W cm^−2^, 8 min). **B** Photothermal heating profiles of HCuS@PDA NPs in aqueous solution (0.5 mg mL^−1^) at different power densities. **C** The absorbance decay of DPBF at 410 nm in the solutions of HCuS@PDA-Ce6/TPP NPs and Ce6 upon laser irradiation (660 nm, 0.3 W cm^−2^, 30 min). **D** The TH302 release profiles of HCuS-TH302@PDA NPs at pH 5.0 or 7.4 with and without laser irradiation at 808 nm (0.5 W cm^−2^, 5 min)

To verify the potential using HCuS@PDA NPs for photothermal therapy, the photothermal behaviors of HCuS NPs and HCuS@PDA NPs were carefully examined with an 808 nm laser. As demonstrated in Fig. 3A, in stark contrast to water, a significant increase in temperature was observed after irradiation for 8 min in both HCuS NPs and HCuS@PDA NPs samples. Moreover, the HCuS NPs-based samples showed a radiant energy-dependent photothermal effect (Fig. 3B and Additional file 1: Fig. S7). It was noting that the temperature of the HCuS@PDA NPs solution (500 μg mL^−1^) rose by 9.7 °C upon irradiation for 8 min even at a low power of 0.5 W cm^−2^. Moreover, the HCuS@PDA NPs solution showed a higher temperature elevation under irradiation than the HCuS NPs solution, suggesting that PDA coating could enhance the photothermal performance of HCuS NPs (Fig. 3B and Additional file 1: Fig. S7). Thereby, HCuS@PDA NPs can be used as the photothermal nanoagents for the following in vitro and in vivo experiments.

The ^1^O_2_ generation capability of HCuS@PDA-Ce6/TPP NPs was chemically detected by using 1,3-diphenylisobenzofuran (DPBF) as the probe. Upon irradiation with a 660 nm laser (0.3 W cm^−2^) for 30 min, no significant change was observed in the absorbance peak of DPBF (Additional file 1: Fig. S8A), indicating the photostability of DPBF. However, in the presence of HCuS@PDA-Ce6/TPP NPs or Ce6, obvious degradation of DPBF was observed with the increase of irradiation time (Fig. 3C and Additional file 1: Fig. S8). These results indicated the effective ^1^O_2_ generation ability of HCuS@PDA-Ce6/TPP NPs.

### In vitro drug release kinetics

Having demonstrated the photothermal and photodynamic performance of our nanoplatform, we next examined the potential of HCuS@PDA NPs as the carrier to encapsulate the chemotherapeutic agents. The controlled release ability of NPs and its pH and NIR dependency were investigated at 37 °C under pH 7.4 and pH 5.0. As shown in Fig. 3D, at pH 7.4, the cumulative release for HCuS-TH302@PDA-Ce6/TPP NPs was 12.12% over the course of 24 h, which was much lower than the drug release at pH 5.0. The result indicated that at physiological pH, the PDA coating was beneficial to avoid the premature release of TH302 during the cycle. In contrast, the loaded TH302 rapidly released under acidic TME, which might be ascribed to that the PDA was partially peeled off the surface of nanoparticles. More attractively, when exposed to a momentary NIR irradiation (808 nm, 0.5 W cm^−2^, 5 min), a burst release of TH302 was detected at both pH 7.4 and pH 5.0. As an external stimulus, the NIR can easily be adjusted remotely in terms of irradiation time and intensity to obtain the required drug release dose. The controlled drug release was beneficial to accumulate high concentrations of drugs at the tumor site and keep them within the treatment window to significantly reduce the side effects chemotherapy and improve the therapeutic efficiency of chemotherapy.

The physiological stability is one of the most critical issues for the application of nanoparticles in biological systems. Therefore, the stability of HCuS@PDA-Ce6/TPP NPs in different solutions including deionized water, PBS buffer (pH 7.4) and cell culture medium (RPMI 1640 supplemented with 10% fetal bovine serum) was investigated. As shown in the DLS results and TEM images (Additional file 1: Fig. S9), the particle size and morphology of HCuS@PDA-Ce6/TPP NPs did not change in all these solutions after standing for 14 days, revealing the good stability of HCuS@PDA-Ce6/TPP NPs in biological systems.

### In vitro mitochondrion targeting ability evaluation of HCuS@PDA-Ce6/TPP NPs

**Fig. 4:**
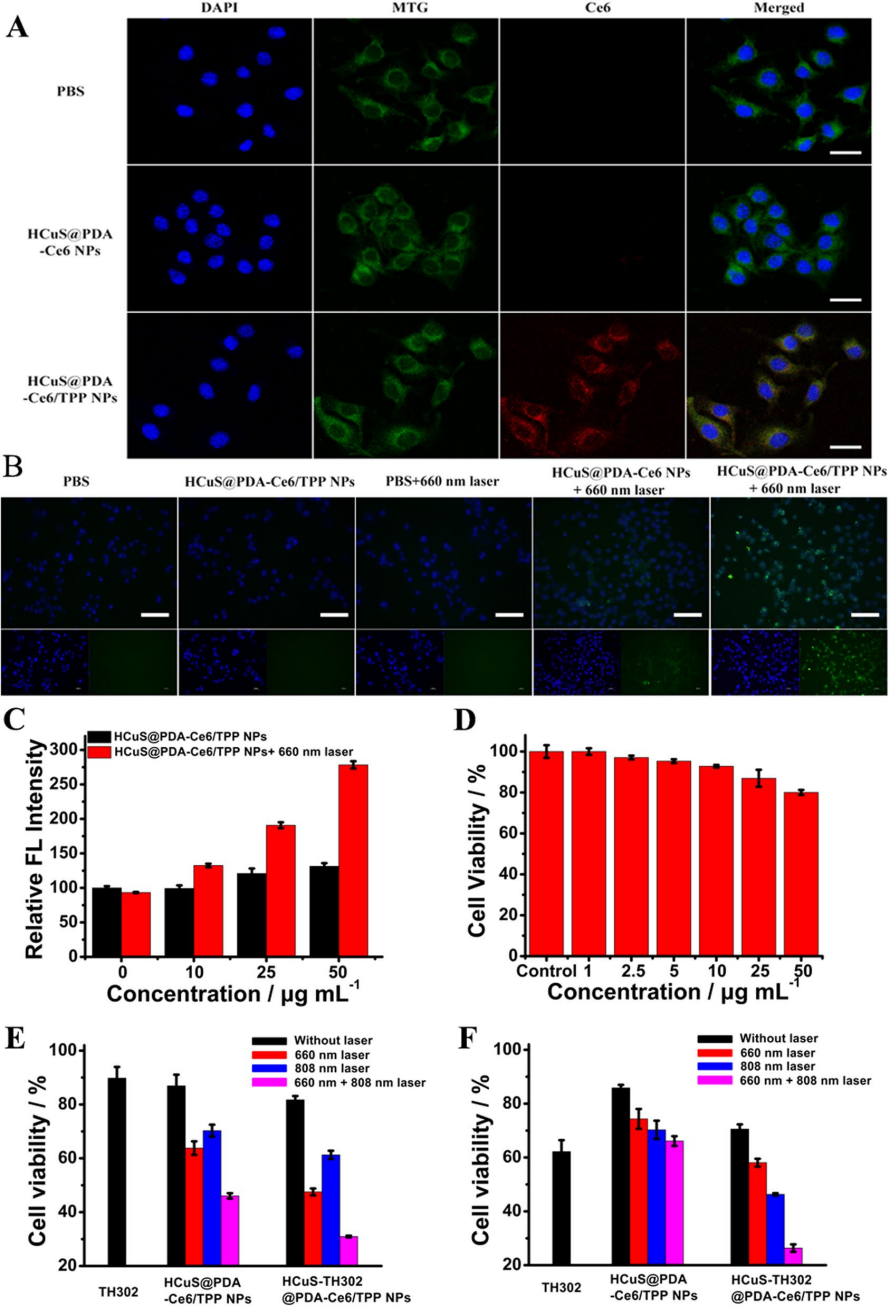
**A** CLSM images showing mitochondrial co-localization of HCuS@PDA-Ce6 NPs and HCuS@PDA-Ce6/TPP NPs in B16F10 cells. Scale bars = 25 μm. **B** Fluorescence microscope images of the cells stained (green) with hypoxia probes Hypoxyprobe-1™, Scale bars = 100 μm. **C** Fluorescence detection of ROS after being treated with HCuS@PDA-Ce6/TPP NPs with and without 660 nm laser irradiation (0.3 W cm^−2^) for 5 min. **D** The cytotoxicity of HCuS@PDA-Ce6/TPP NPs on B16F10 cells. **E** In vitro cytotoxicity of TH302, HCuS@PDA-Ce6/TPP NPs and HCuS-TH302@PDA-Ce6/TPP NPs with laser irradiation in normoxia condition. **F** In vitro cytotoxicity of TH302, HCuS@PDA-Ce6/TPP NPs and HCuS-TH302@PDA-Ce6/TPP NPs with laser irradiation in hypoxia condition

The colocalization analysis was utilized to observe the mitochondria targeting ability of HCuS@PDA-Ce6/TPP NPs. The B16F10 cells were incubated with HCuS@PDA-Ce6/TPP NPs for 4 h, and then stained with DAPI and Mito-Tracker Green, a commercial mitochondria-staining dye. As shown in Fig. 4A, fluorescence signals from the HCuS@PDA-Ce6/TPP NPs (Ce6 channel) and Mito-Tracker Green were overlapped well in B16F10 cells, indicating that HCuS@PDA-Ce6/TPP NPs were selectively localized in mitochondria. In contrast, the HCuS@PDA-Ce6 NPs treated cells were substantially different, with little overlap of the fluorescent signals of Mito-Tracker and the NPs. Together, these results demonstrated that TPP decoration endowed the HCuS@PDA-Ce6/TPP NPs with selective mitochondrial targeting function, which could greatly improve the efficiency of tumor phototherapy.

### In vitro ROS/hypoxia assay

Ce6 as a photosensitizer can consume O_2_ to generate ROS under 660 nm laser irradiation, which in turn exaggerates tumor hypoxia and improves the therapeutic effect of TH302 [6, 45]. Before exploration of the biomedical application of HCuS@PDA-Ce6/TPP NPs, we first determined the nanoplatform-mediated light-triggered intracellular ROS generation and hypoxia levels using DCFH-DA [46] and Hypoxyprobe-1™ [6] as the indicators, respectively. Figure 4C showed that the 660 nm laser irradiation (0.3 W cm^−2^, 5 min) itself could not increase the ROS level in B16F10 cells. However, in comparison with the ROS levels of the untreated group and HCuS@PDA-Ce6/TPP NPs treated group without laser irradiation, the HCuS@PDA-Ce6/TPP NPs treated cells with laser irradiation exhibited significant high levels of ROS in a dose-dependent manner. Furthermore, the fluorescence microscope (Fig. 4B) results showed that HCuS@PDA-Ce6/TPP NPs-mediated PDT consumed O_2_ to generate ^1^O_2_, which further led to hypoxia. In order to assess the role of PDT in inducing hypoxia, we also conducted a number of control experiments. As shown in Fig. 4B, in normoxia condition, HCuS@PDA-Ce6/TPP NPs could not induce tumor hypoxia without laser irradiation. Moreover, compared to the control cells, there was no change in O_2_ consumption in PBS treated cells even upon laser irradiation, indicating that the PDT process relied on Ce6 molecules which indeed aggravated intracellular hypoxia. The exacerbated hypoxia in tumor cells can greatly facilitate the therapeutic effect of TH302.

### In vitro synergistic therapeutic effect

The cytotoxicity of the HCuS@PDA-Ce6/TPP NPs to B16F10 cells was also evaluated to eliminate any non-specific effect. As shown in Fig. 4D, the viability of B16F10 cells remained above 80% even after incubation with 50 µg mL^−1^ HCuS@PDA-Ce6/TPP NPs for 24 h. This clearly indicated that the synthesized HCuS@PDA-Ce6/TPP NPs exhibited good biocompatibility and showed no obvious toxicity to normal tissues and cells.

The efficient photodynamic and photothermal preformance encourged us to investigate the phototherapeutic capability of HCuS@PDA-Ce6/TPP NPs against B16F10 cells by MTT assay. The efficiency of PTT and PDT can be precisely controlled by changing the light irradiation power with different lasers. As shown in Additional file 1: Fig. S10, for PDT, at the concentration of 50 µg mL^−1^, the cell viability reduced to 49.28% with laser power density as 0.3 W cm^−2^, indicating HCuS@PDA-Ce6/TPP NPs had an efficient PDT effect even at a low laser power (0.3 W cm^−2^). While for PTT, although HCuS@PDA-Ce6/TPP NPs displayed a certain degree of photothermal effect at a low laser power (0.5 W cm^−2^), the therapeutic efficacy was lower than that with a laser power density of 1.0 W cm^−2^. Therefore, considering the therapeutic effects for PDT and PTT, 660 nm at 0.3 W cm^−2^ and 808 nm at 1.0 W cm^−2^ were chosen as the laser irradiation conditions for the following therapeutic study.

Subsequently, the synergistic effects of PTT and PDT were investigeted. B16F10 cells were incubated with different concentrations of HCuS@PDA-Ce6/TPP NPs for 4 h, and then PDT, PTT, and PDT/PTT synergistic treatments were perfomed. As shown in Additional file 1: Fig. S10B, PDT, PTT, and their synergism effects all exhibited a concentration-dependent manner in cancer cell killing. At the concentration of 25 μg mL^−1^, the cell viabilities of HCuS@PDA-Ce6/TPP NPs-treated cells were 70.28% and 63.80% after single photothermal and photodynamic treatment, respectively. However, the cytotoxicity was significantly enhanced by the synergistic PDT/PTT, which decreased the cell viability to 46.05%.

Considering the oxygen levels in TME and the fact that PDT and the chemotherapy of TH302 were oxygen dependent, the in vitro cytotoxicity of HCuS-TH302@PDA-Ce6/TPP NPs against B16F10 cells was studied in hypoxic (1% O_2_) and normoxic (21% O_2_) atmosphere [47]. As expected, TH302 and the nanoplatform exhibited oxygen level related cytotoxicity. In normoxic atmosphere (Fig. 4E), negligible toxicity was observed for TH302 alone (cell viability of 89.77%). On the contrary, cell viability with HCuS@PDA-Ce6/TPP (25 µg mL^−1^)-mediated PDT was 63.80%, while, HCuS-TH302@PDA-Ce6/TPP NPs reduced the cell viability to 47.52% under the photodynamic condition due to light-triggered hypoxia-activated synergistic PDT-chemotherapy. Moreover, with a combination of 660 and 808 nm laser irradiation, the cell viability was only 30.94%, which was quite lower than that of PDT and PTT alone. While, under the hypoxic condition (Fig. 4F), HCuS-TH302@PDA-Ce6/TPP NPs exhibited lower cytotoxicity compared with free TH302 at equivalent TH302 concentration, which may arise from the sustained release effect of HCuS-TH302@PDA-Ce6/TPP NPs. The cell viability decreased significantly with 808 nm laser irradiation, owing to the increased release of TH302 and photothermal effect (Fig. 4F). Regardless of the possible limited PDT effect under hypoxic environment, HCuS-TH302@PDA-Ce6/TPP NPs displayed the highest cytotoxicity upon a combination of 660 and 808 nm laser irradiation, demonstrating that this combined PTT, oxygen-consumed PDT and the successive bioreductive chemotherapy had the optimum therapeutic efficacy.

### In vitro and in vivo biocompatibility experiment

**Fig. 5:**
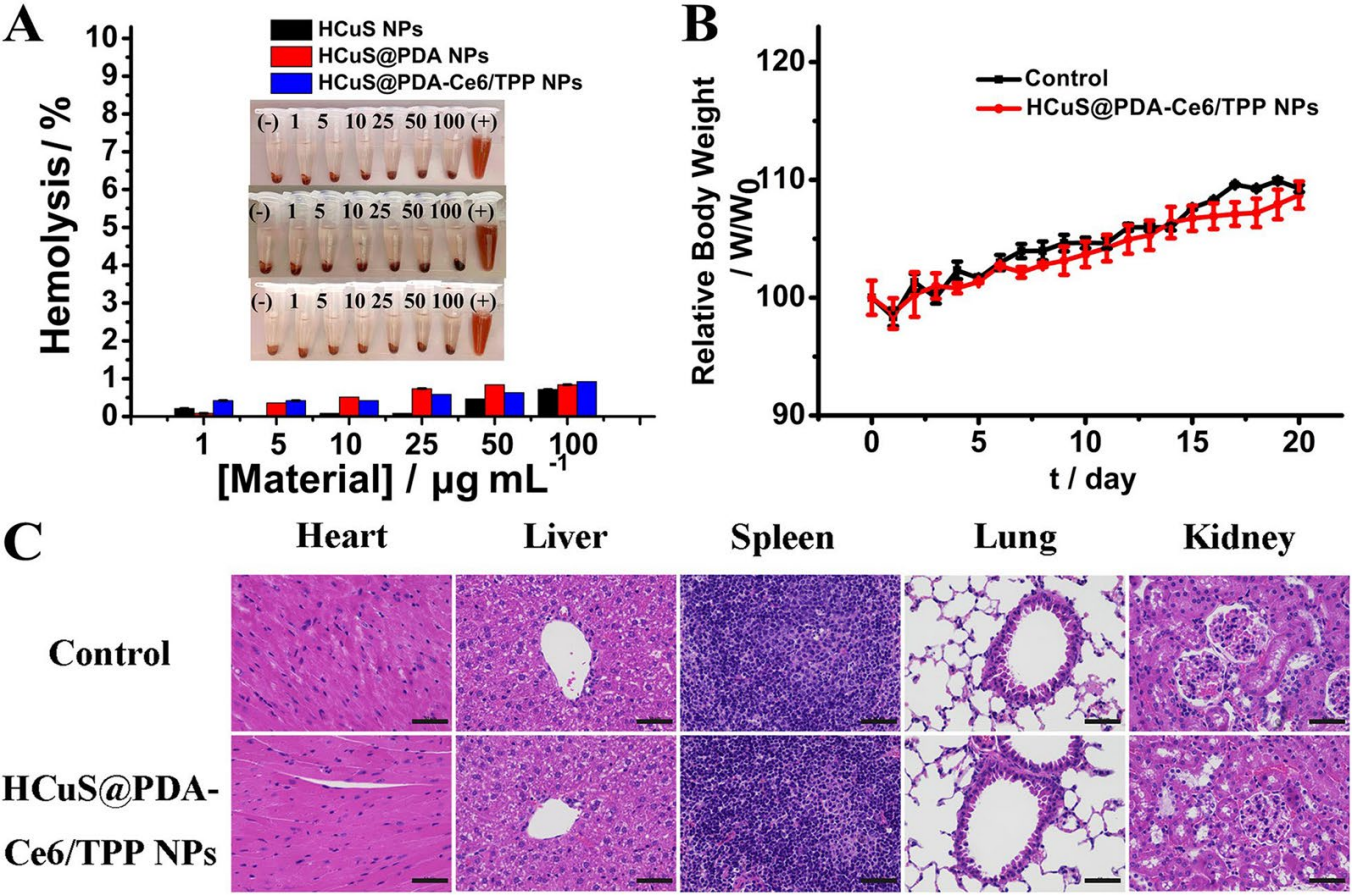
The biocompatibility of HCuS-based nanomaterials. **A** Hemolysis assays for HCuS NPs, HCuS@PDA NPs and HCuS@PDA-Ce6/TPP NPs. The concentration of nanomaterials varied from 1 to 100 μg mL^−1^ (mean ± SD, n = 3 for each sample). **B** The relative body weights with different treatment from tumor-free mice (mean ± SD, n = 6 for each sample). **C** H&E-stained images of main organ slices collected from tumor-free mice of different groups. Scale bars = 50 μm

To ensure the possibility and safety for cancer treatment, it is of importance to study the in vitro and in vivo biocompatibility of the nanoplatform. Hemolysis testing as a classical assay was employed for evaluating the hemocompatibility. As shown in Fig. 5A, within the range of 1–100 μg mL^−1^, HCuS@PDA-Ce6/TPP NPs only caused < 2% hemolysis, which was considered to be biocompatible in accordance with ISO/TR 7406 (the permissible limit for hemolysis is 5%) [48]. The in vivo biocompatibility of HCuS@PDA-Ce6/TPP NPs was also analyzed by the change of body weight after injecting the nanomaterials (Fig. 5B). No significant difference in body weight was observed between nanomaterials-treated mice and untreated mice within 21 days. On day 21, the main organs of the mice were collected and examined by hematoxylin–eosin (H&E) staining (Fig. 5C). H&E-stained pathological sections of the main organs of nanomaterials treated mice including hearts, livers, spleens, lungs and kidneys exhibited no apparent lesions or abnormalities as compared with that from the untreated group, suggesting the excellent in vivo biocompatibility of our nanoplatform.

### In vivo antitumor effects

**Fig. 6:**
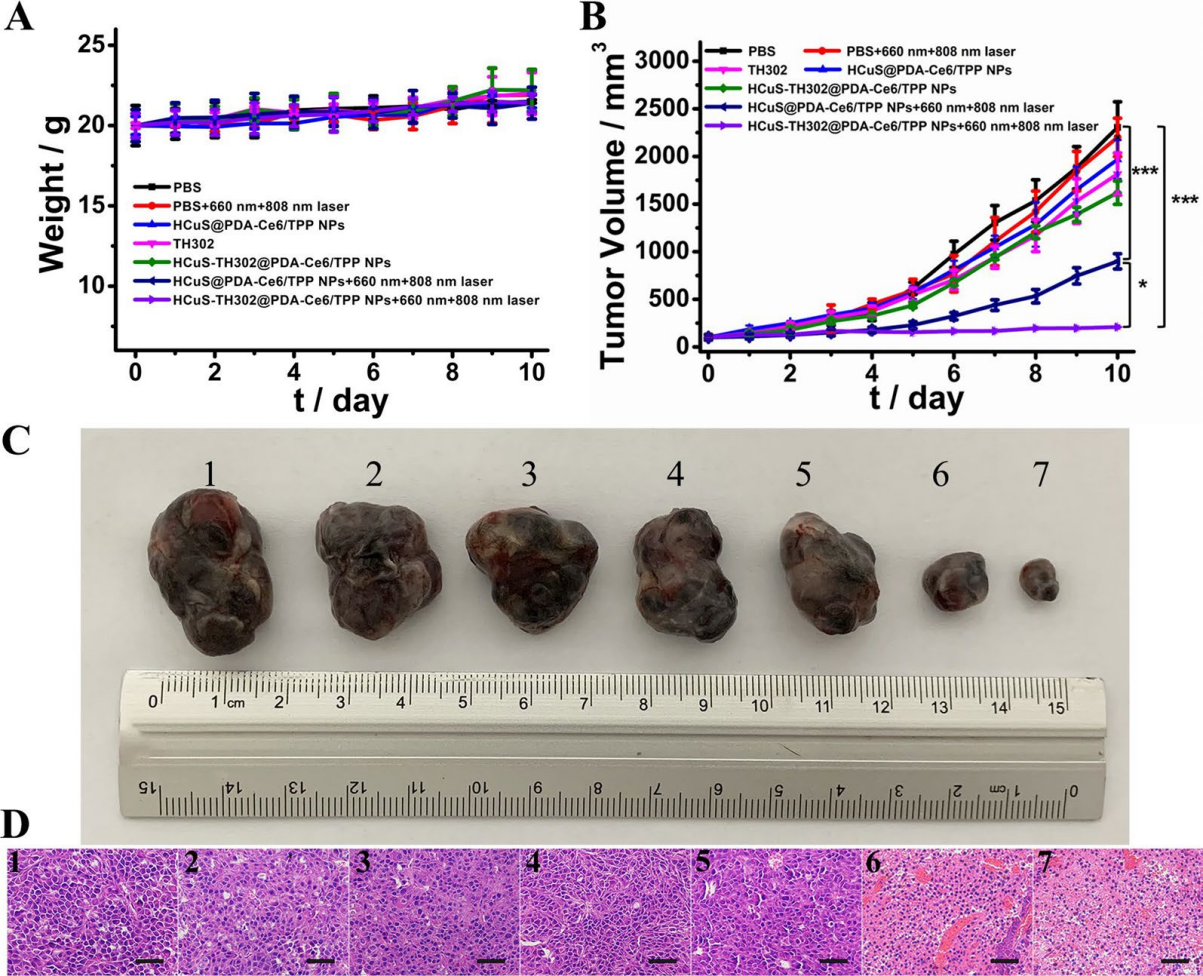
In vivo antitumor efficacy of different samples in C57BL/6 mice bearing B16F10 cells. **A** The average body weight variations during the period of treatment within 10 days. **B** The relative tumor volumes after different treatment within 10 days. (n = 7, *P < 0.05, **P < 0.01, ***P < 0.001). **C** Photographs of the sacrificed tumor tissues after various treatment. **D** The H&E staining images of the dissected tumor tissues after 10 days of treatment. Scale bar = 50 µm. Note that 1–7 represents mice groups with different treatments: (1) PBS, (2) PBS + 660 nm + 808 nm laser, (3) HCuS@PDA-Ce6/TPP NPs, (4) TH302, (5) HCuS-TH302@PDA-Ce6/TPP NPs, (6) HCuS@PDA-Ce6/TPP NPs + 660 nm + 808 nm laser, (7) HCuS-TH302@PDA-Ce6/TPP NPs + 660 nm + 808 nm laser

To determine the anticancer outcomes of different treatment strategies, B16F10 tumor-bearing mice were randomly divided into seven groups: (1) PBS, (2) PBS + 660 nm + 808 nm laser, (3) HCuS@PDA-Ce6/TPP NPs, (4) TH302, (5) HCuS-TH302@PDA-Ce6/TPP NPs, (6) HCuS@PDA-Ce6/TPP NPs + 660 nm + 808 nm laser, (7) HCuS-TH302@PDA-Ce6/TPP NPs + 660 nm + 808 nm laser. Mice were injected with free drugs or nanomaterials on day 0. After injection for 4 h [49], tumor tissues from the NIR-involved groups were exposed to 660 nm laser irradiation (0.3 W cm^−2^, 5 min) and 808 nm laser irradiation (1.0 W cm^−2^, 5 min), respectively. Mice receiving different treatments showed no significant changes in body weight variations compared to the PBS control group, indicating that all the treatments could be well tolerated (Fig. 6A). The normalized average tumor volumes as a function of treatment time were presented in Fig. 6B. As expected, the tumor volume of the control group increased throughout the study. Laser irradiation itself did not slow down this growth trend. Administration of TH302 and HCuS-TH302@PDA-Ce6/TPP NPs could partially inhibit the tumor growth with tumor growth inhibition (TGI) rates of 22.27% and 31.34% respectively. Using HCuS@PDA-Ce6/TPP NPs upon 660 nm and 808 nm laser irradiation which combined PDT and PTT showed the moderated growth inhibition effect with TGI of 61.04%. Importantly, compared to other groups, the TGI (95.53%) was significantly increased in the group of HCuS-TH302@PDA-Ce6/TPP NPs plus 660 nm and 808 nm laser irradiation, which integrated the chemotherapy, PTT and PDT.

The representative images of tumors were shown in Fig. 6C. The H&E staining was also employed to determine the cells apoptosis of different groups (Fig. 6D). The results revealed that the treatment of HCuS-TH302@PDA-Ce6/TPP NPs plus laser irradiation could significantly induce tumor cell apoptosis and ablate tumors, indicating that multimodal synergistic therapy was more effective than a single treatment. These data were in accordance with the results observed in the experiment of tumor growth. Besides, the biodistribution of HCuS@PDA-Ce6/TPP NPs in the main tissues (heart, liver, spleen, lung, kidney, and tumor) after intravenous injection was also investigated by ICP-AES. As indicated in Additional file 1: Fig. S11, the accumulation of HCuS@PDA-Ce6/TPP NPs in tumor tissues were 11.78 ± 0.20% and 16.01 ± 0.10% ID/g at 4 h and 8 h after injection, respectively. Therefore, the HCuS@PDA-Ce6/TPP NPs with effective tumor accumulation could achieve tumor-targeted therapy.

## Conclusions

In summary, a new class of mitochondria-targeting therapeutic system based on HCuS-TH302@PDA-Ce6/TPP NPs was proposed for tumor-specific synergistic hypoxia-activated chemotherapy/PDT/PTT. Benefit from the excellent photothermal conversion property, photodynamic performance as well as the specifically precise targeting capability, the nanoplatform can efficiently accumulate in mitochondria in cancer cells and then induce apoptotic hyperthermia and generate sufficient ROS upon laser irradiation. Furthermore, once accumulated in tumor, the nanoplatform responded to the TME conditions and laser irradiation, followed by release of TH302. The consumption of O_2_ in the PDT process indeed aggravated tumor hypoxia, which greatly facilitated the therapeutic effect of the released TH302, leading to a synergistic anticancer effect of hypoxia activated chemotherapy, PTT and PDT. Our work would promote the clinical application of the combination of HAPs plus phototherapy in tumor treatment. Considering the wider applications in the future, further studies are planned to develop more efficient photosensitizer to construct single NIR-laser induced multifunctional nanoplatform for synergistic cancer therapy.

## Supplementary Information

**Additional file 1: Figure S1.** SEM images of (A) HCuS NPs, (B) HCuS@PDA NPs, (C) HCuS@PDA-Ce6 NPs and (D) HCuS@PDA-Ce6/TPP NPs. Scale bars = 200 nm. **Figure S2.** N_2_ absorption/desorption isotherms of HCuS NPs. **Figure S3.** Zeta potentials of HCuS-based nanomaterials. **Figure S4.** Size measurement results (average values) of (A) HCuS NPs, (B) HCuS@PDA NPs, (C) HCuS@PDA-Ce6 NPs and (D) HCuS@PDA-Ce6/TPP NPs as measured with DLS. **Figure S5.** Thermogravimetric analysis (TGA) curves of HCuS NPs and HCuS@PDA NPs. **Figure S6.** TEM images of (A) HCuS@PDA-Ce6 NPs and (B) HCuS@PDA-Ce6/TPP NPs. Scale bars = 100 nm. **Figure S7.** Photothermal heating profiles of HCuS NPs (0.5 mg mL^−1^) in aqueous solution at different power densities. **Figure S8.** Detection of singlet oxygen generation using DPBF as the probe. Time dependent absorption spectra of DPBF in (A) PBS, (B) Ce6, (C) HCuS@PDA-Ce6/TPP NPs solutions under 660 nm laser irradiation (0.3 W cm^−2^). **Figure S9.** The physiological stability of HCuS@PDA-Ce6/TPP NPs in different solutions. (A) DLS studies of HCuS@PDA-Ce6/TPP NPs in different solutions. The TEM images of HCuS@PDA-Ce6/TPP NPs incubated in (B) deionized water, (C) PBS buffer (pH 7.4) and (D) cell culture medium (RPMI 1640 medium with 10% fetal bovine serum) after standing for 14 days. Scale bars = 100 nm. **Figure S10.** Relative viability of B16F10 cells treated with HCuS@PDA-Ce6/TPP NPs under (A) 0.3 W cm^−2^ for 660 nm; 0.5 W cm^−2^ for 808 nm and (B) 0.3 W cm^−2^ for 660 nm; 1.0 W cm^−2^ for 808 nm laser irradiation. **Figure S11.** The biodistribution of HCuS@PDA-Ce6/TPP NPs after intravenous injection by ICP-AES assay (n = 4).

## Data Availability

All data are available in the main text or the additional materials.

## Abbreviations

HAPs: Hypoxia-activated prodrugs
PDT: Photodynamic therapy
PTT: Photothermal therapy
PDA: Polydopamine
Ce6: Chlorin e6
TPP: (5-carboxypentyl) triphenyl phosphonium
TME: Tumor microenvironment
PS: Photosensitizer
ROS: Reactive oxygen species
NIR: Near-infrared
Tris: Tris(hydroxymethyl)aminomethane
NHS: *N*-Hydroxysuccinimide
NaOH: Sodium hydroxide
EDC: 1-(3-Dimethylaminopropyl)-3-ethylcarbodiimide hydrochloride
DCFH-DA: 2′,7′-Dichlorofluorescein diacetate
MTT: 3-(4,5-Dimethylthiazol-2-yl)-2,5-diphenyltetrazolium bromide
DAPI: 4′,6-Diamidino-2-phenylindole
HRTEM: High resolution transmission electron microscopy
XRD: X-ray diffraction
BET: Brunauer–Emmett–Teller
FTIR: Fourier translation infrared
HPLC: High performance liquid chromatography
FBS: Fetal bovine serum
RBCs: Red blood cells
TGI %: Tumor growth inhibition rate
ANOVA: One-way analysis of variance
TGA: Thermogravimetric analysis
DPBF: 1,3-Diphenylisobenzofuran
