# Supplementary material for: Mitochondria-targeting multifunctional nanoplatform for cascade phototherapy and hypoxia-activated chemotherapy
Source: J Nanobiotechnology. 2022 Jan 21;20:42. doi: 10.1186/s12951-022-01244-9 (PMC8780403; doi:10.1186/s12951-022-01244-9)
Supplement: Supplementary file 1 — Additional file 1: Figure S1. SEM images of (A) HCuS NPs, (B) HCuS@PDA NPs, (C) HCuS@PDA-Ce6 NPs and (D) HCuS@PDA-Ce6/TPP NPs. Scale bars = 200 nm. Figure S2. N2 absorption/desorption isotherms of HCuS NPs. Figure S3. Zeta potentials of HCuS-based nanomaterials. Figure S4. Size measurement results (average values) of (A) HCuS NPs, (B) HCuS@PDA NPs, (C) HCuS@PDA-Ce6 NPs and (D) HCuS@PDA-Ce6/TPP NPs as measured with DLS. Figure S5. Thermogravimetric analysis (TGA) curves of HCuS NPs and HCuS@PDA NPs. Figure S6. TEM images of (A) HCuS@PDA-Ce6 NPs and (B) HCuS@PDA-Ce6/TPP NPs. Scale bars = 100 nm. Figure S7. Photothermal heating profiles of HCuS NPs (0.5 mg mL−1) in aqueous solution at different power densities. Figure S8. Detection of singlet oxygen generation using DPBF as the probe. Time dependent absorption spectra of DPBF in (A) PBS, (B) Ce6, (C) HCuS@PDA-Ce6/TPP NPs solutions under 660 nm laser irradiation (0.3 W cm−2). Figure S9. The physiological stability of HCuS@PDA-Ce6/TPP NPs in different solutions. (A) DLS studies of HCuS@PDA-Ce6/TPP NPs in different solutions. The TEM images of HCuS@PDA-Ce6/TPP NPs incubated in (B) deionized water, (C) PBS buffer (pH 7.4) and (D) cell culture medium (RPMI 1640 medium with 10% fetal bovine serum) after standing for 14 days. Scale bars = 100 nm. Figure S10. Relative viability of B16F10 cells treated with HCuS@PDA-Ce6/TPP NPs under (A) 0.3 W cm−2 for 660 nm; 0.5 W cm−2 for 808 nm and (B) 0.3 W cm−2 for 660 nm; 1.0 W cm−2 for 808 nm laser irradiation. Figure S11. The biodistribution of HCuS@PDA-Ce6/TPP NPs after intravenous injection by ICP-AES assay (n = 4). [file 12951_2022_1244_MOESM1_ESM.docx]

**Mitochondria-targeting multifunctional nanoplatform for cascade phototherapy and hypoxia-activated chemotherapy**

Jie Lv, Shuangling Wang, Duo Qiao, Yulong Lin, Shuyang Hu and Meng Li*

*Correspondence: limeng87@hotmail.com

College of Pharmacy, Key Laboratory of Innovative Drug Development and Evaluation, Hebei Medical University, Shijiazhuang, 050017, China


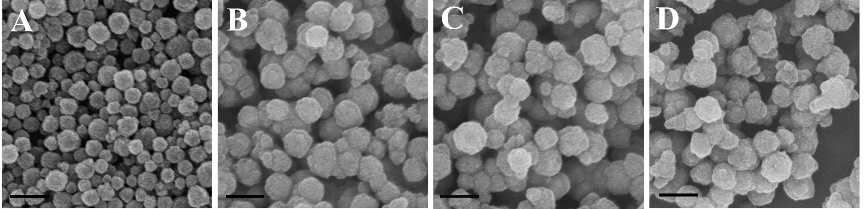


**Fig. S1.** SEM images of (A) HCuS NPs, (B) HCuS@PDA NPs, (C) HCuS@PDA-Ce6 NPs and (D) HCuS@PDA-Ce6/TPP NPs. Scale bars = 200 nm.

**
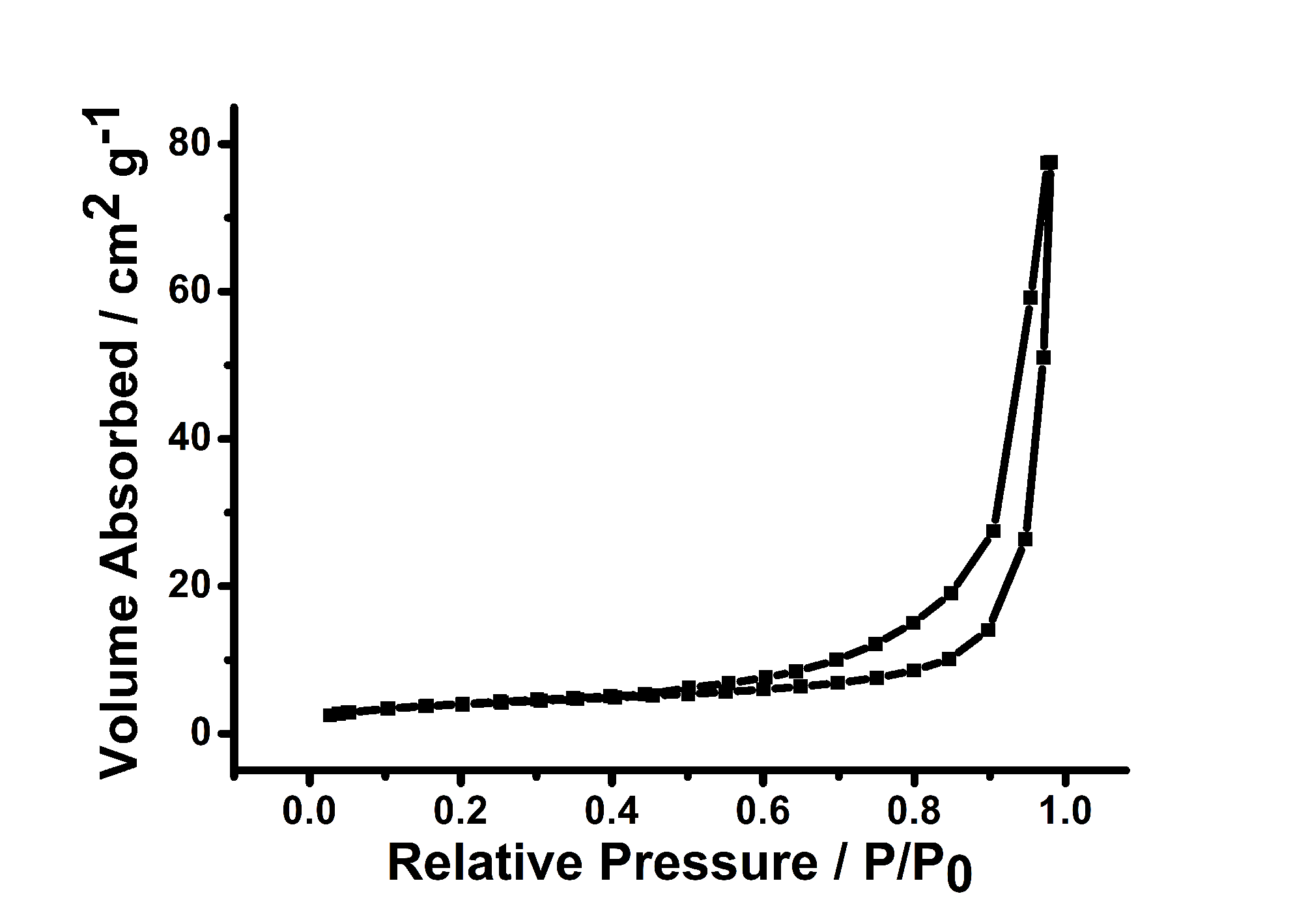
**

**Fig. S2.** N_2_ absorption/desorption isotherms of HCuS NPs.





**Fig. S3.** Zeta potentials of HCuS-based nanomaterials.





**Fig. S4.** Size measurement results (average values) of (A) HCuS NPs, (B) HCuS@PDA NPs, (C) HCuS@PDA-Ce6 NPs and (D) HCuS@PDA-Ce6/TPP NPs as measured with DLS.





**Fig. S5.** Thermogravimetric analysis (TGA) curves of HCuS NPs and HCuS@PDA NPs.


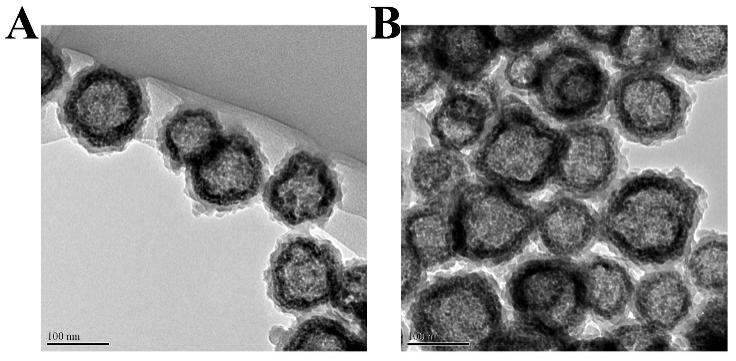


**Fig. S6.** TEM images of (A) HCuS@PDA-Ce6 NPs and (B) HCuS@PDA-Ce6/TPP NPs. Scale bars = 100 nm.


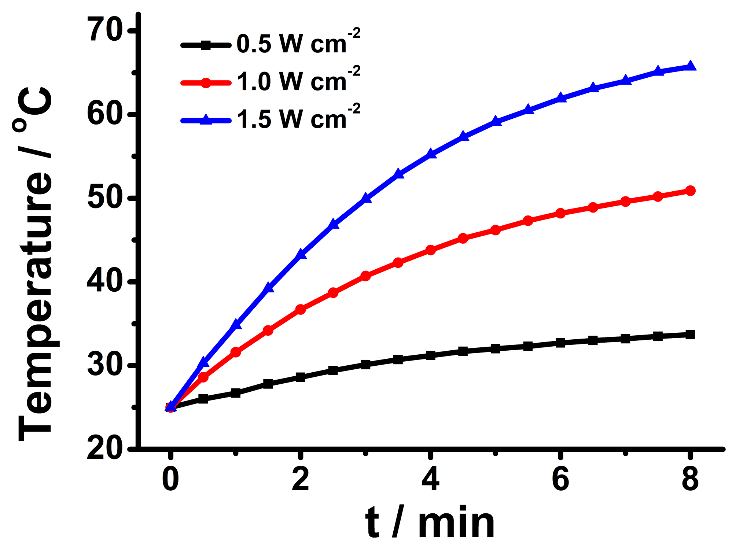


**Fig. S7.** Photothermal heating profiles of HCuS NPs (0.5 mg mL^-1^) in aqueous solution at different power densities.


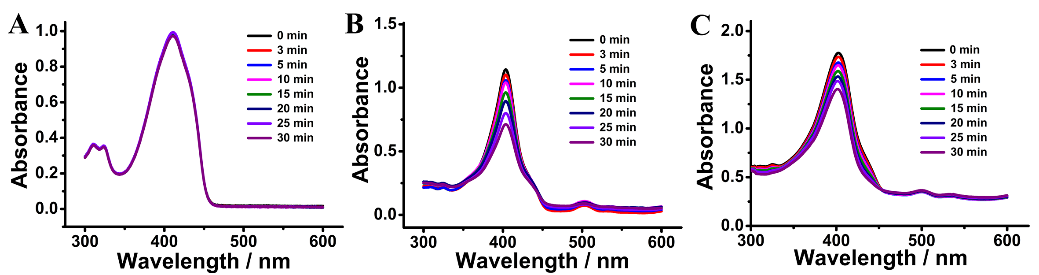


**Fig. S8.** Detection of singlet oxygen generation using DPBF as the probe. Time dependent absorption spectra of DPBF in (A) PBS, (B) Ce6, (C) HCuS@PDA-Ce6/TPP NPs solutions under 660 nm laser irradiation (0.3 W cm^-2^).


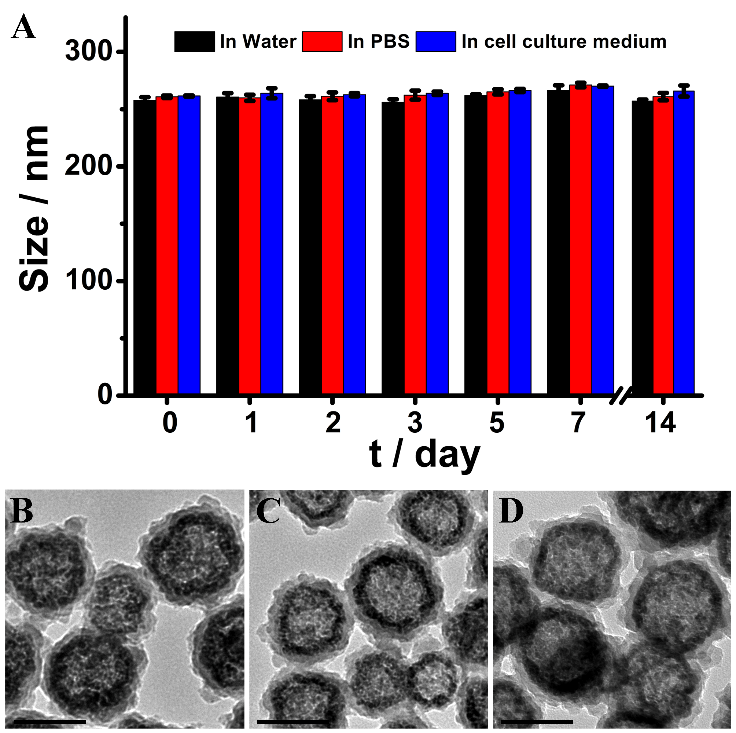


**Fig. S9.** The physiological stability of HCuS@PDA-Ce6/TPP NPs in different solutions. (A) DLS studies of HCuS@PDA-Ce6/TPP NPs in different solutions. The TEM images of HCuS@PDA-Ce6/TPP NPs incubated in (B) deionized water, (C) PBS buffer (pH 7.4) and (D) cell culture medium (RPMI 1640 medium with 10% fetal bovine serum) after standing for 14 days. Scale bars = 100 nm.


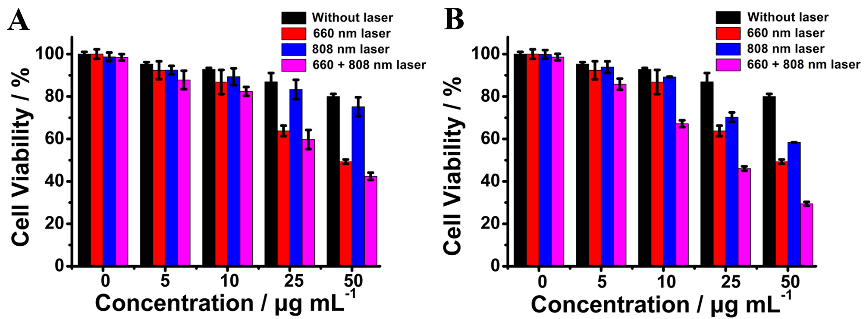


**Fig. S10.** Relative viability of B16F10 cells treated with HCuS@PDA-Ce6/TPP NPs under (A) 0.3 W cm^-2^ for 660 nm; 0.5 W cm^-2^ for 808 nm and (B) 0.3 W cm^-2^ for 660 nm; 1.0 W cm^-2^ for 808 nm laser irradiation.





**Fig. S11.** The biodistribution of HCuS@PDA-Ce6/TPP NPs after intravenous injection by inductively coupled plasma-atomic emission spectrometer (ICP-AES) assay (n = 4).
